# Black Pleural Effusion as a Complication of Acute Pancreatitis

**DOI:** 10.7759/cureus.32783

**Published:** 2022-12-21

**Authors:** Antony J Arumairaj, Fidencio Davalos, Hussein Matari, Abayomi O Bamgboje, Imnett Habtes

**Affiliations:** 1 Internal Medicine, New York Medical College, Metropolitan Hospital Center, New York, USA; 2 Pulmonary Critical Care, New York Medical College, Metropolitan Hospital Center, New York, USA; 3 Radiology, New York Medical College, Metropolitan Hospital Center, New York, USA; 4 Pulmonary and Critical Care, New York Medical College, Metropolitan Hospital Center, New York, USA

**Keywords:** pancreatic pseudocyst, pancreatic pleural effusion, pancreatic ascites, black pleural effusion, chronic pancreatitis, acute pancreatitis

## Abstract

Transient and reactive pleural effusion is a known consequence of acute pancreatitis. Usually, the pleural effusion is unilateral, transudate, straw-colored, and self-resolving. We report a rare case of massive left-sided black pleural effusion as a complication of acute pancreatitis with the background of chronic pancreatitis being secondary to alcohol abuse. The pleural effusion resulted in hypoxic respiratory failure. However, the patient had significant improvement after drainage of the pleural effusion and the appropriate management of sepsis with broad-spectrum antibiotics. The patient had a significant improvement and recovery with conservative management without the need for endoscopic therapy or surgical intervention.

## Introduction

Black pleural effusions are extremely rare presentations and have been reported previously in only a handful of patients with lung malignancy, metastatic melanoma, pancreatic-pleural fistula, rupture of the pancreatic pseudocyst, biliopleural fistula, fungal infections of Aspergillus niger and Rhizopus oryzae [1,2]. Identifying the etiology of the pleural effusion and initiating appropriate management are crucial to preventing fatal complications such as respiratory failure and recurrent accumulation of pleural effusions. We present a rare case of a 48-year-old male patient with large left-sided black pleural effusion secondary to acute pancreatitis and pancreatic pseudocysts who had a remarkable improvement with the timely administration of thoracentesis and pleural drainage. This case report was presented as an abstract at the CHEST conference on October 18, 2020.

## Case presentation

**Figure 1:**
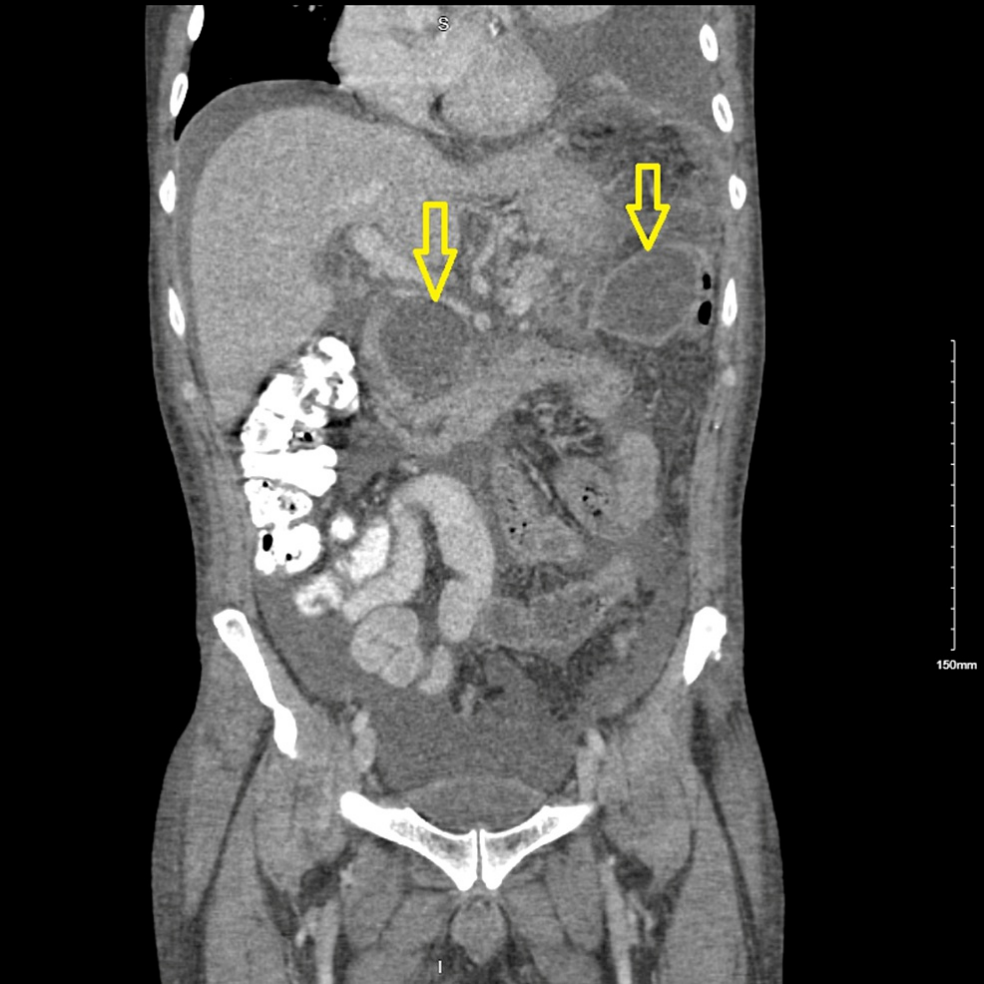
Coronal section of abdomen CT demonstrating ascites and pancreatic pseudocysts in the head and tail of pancreas, indicated with arrow marks.

**Figure 2:**
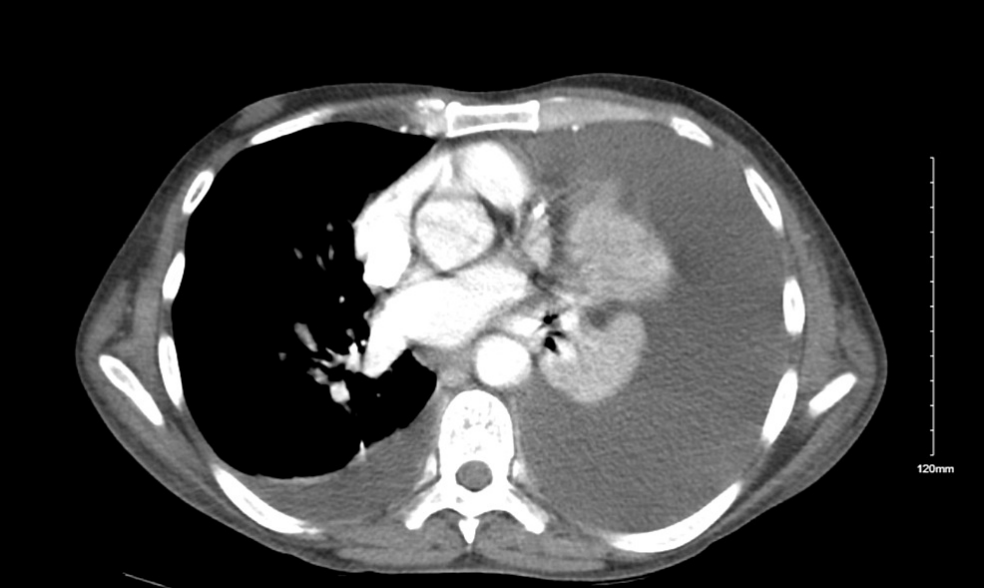
Axial section of chest CT demonstrating a massive left-sided pleural effusion and a moderate right-sided pleural effusion with compression atelectasis.

A 48-year-old male with a history of chronic pancreatitis secondary to chronic alcohol abuse presented to the emergency department with complaints of two days of shortness of breath and epigastric pain radiating to his back and left shoulder after consuming alcohol. His serum amylase was elevated to 1774 units per liter (U/L), and his serum lipase was elevated to 1122 U/L. Abdomen computer tomography (CT) showed peripancreatic edema suggestive of acute pancreatitis that had an interval increase of a distal pancreatic pseudocyst measuring 6 cm, stable pseudocyst at the head of the pancreas, pancreatic calcifications, and large ascites (Figure 1). Chest CT demonstrated a massive left pleural effusion and a moderate right pleural effusion (Figure 2).

**Figure 3:**
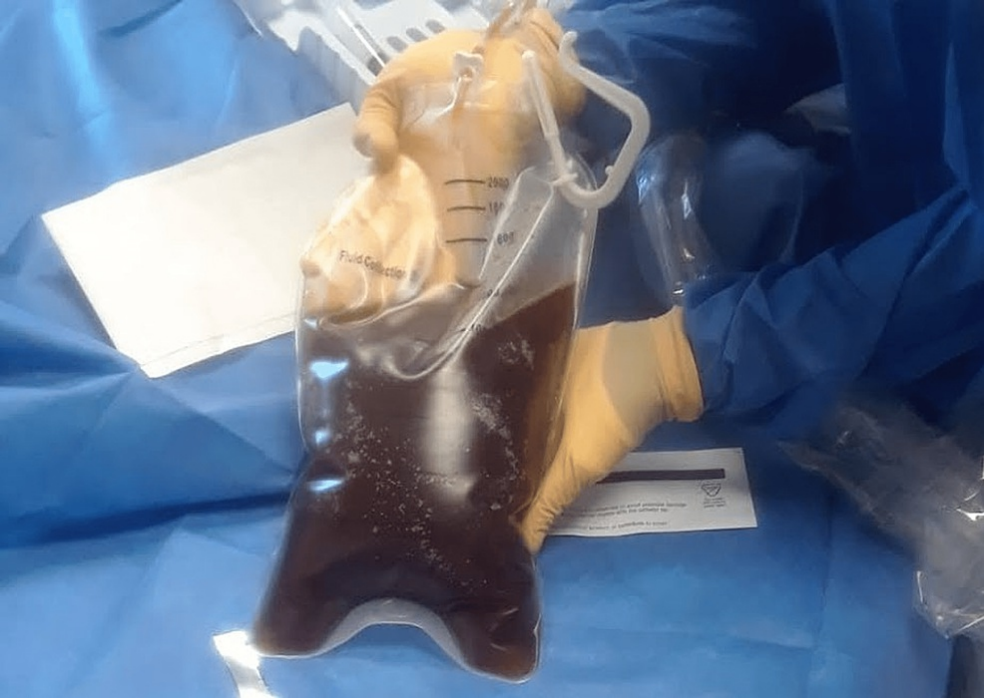
Image of the black pleural fluid collected in the pleural drainage bag with thoracocentesis.

Paracentesis was performed. Ascitic fluid analysis was consistent with bacterial peritonitis, and to treat bacterial peritonitis, the patient was started on intravenous fluid hydration and ceftriaxone. Ascitic fluid cultures were negative. The patient continued to experience severe abdominal pain and developed a fever with a temperature of 101.8 Fahrenheit, tachycardia with a heart rate of 121 beats per minute, and tachypnea with a respiratory rate of 34 per minute. He was transferred to the intensive care unit for the management of sepsis from acute pancreatitis complicated by bacterial peritonitis and acute hypoxic respiratory failure due to bilateral pleural effusions. Antibiotics were escalated to zosyn and vancomycin to broaden the antimicrobial coverage. The patient underwent a pigtail chest tube insertion on the left side, draining 1,300 ml of black pleural fluid on day one (Figure 3). The patient’s respiratory status improved significantly over the next 48 hours, and he no longer required oxygen supplementation.

The pleural fluid analysis was indicative of an exudative pleural effusion. Pleural fluid white cell count was elevated to 2,342/μL with a neutrophilic predominance of 92%. Pleural fluid amylase was grossly elevated to 3,434 U/L, and pleural fluid lipase was elevated to 9,200 U/L. These values signified that the black pleural effusion was secondary to underlying acute pancreatitis. Pleural fluid bacterial and fungal cultures were negative. Drainage of the black pleural fluid using the pigtail chest tube was continued, draining 5,000 ml of black pleural fluid. The patient improved hemodynamically with the resolution of the sepsis and drainage of the massive left pleural effusion. He was then transferred to a tertiary care hospital for endoscopic management of the pancreatic pseudocyst. He underwent repeat chest CT and magnetic resonance cholangiopancreatography (MRCP), both of which showed no evidence of pancreatic-pleural fistula. After pleural effusions resolved, the chest tube was removed. The patient subsequently underwent upper gastrointestinal (GI) endoscopic ultrasound-guided fine-needle aspiration of the pancreatic pseudocyst. The culture of the pseudocyst fluid was negative. Upper GI endoscopy was unremarkable, except for an erythematous duodenum. The patient improved symptomatically with the completion of the intravenous antibiotic course. There was no recurrence of pleural effusions.

## Discussion

Pancreatic ascites and pancreatic-pleural effusion are rare complications of acute and chronic pancreatitis [3]. Acute and chronic pancreatitis disrupts the pancreatic duct, resulting in the leakage of pancreatic secretions into the peritoneal cavity [4]. When the amylase-rich pancreatic secretions enter the peritoneal cavity, it produces severe inflammation leading to an outpouring of albumin, which results in pancreatic ascites [5,6]. Black pleural effusions as a sequela of pancreatic ascites are an extremely rare presentation [7]. 

In acute pancreatitis, the acute inflammatory reaction results in the walling-off of the pancreatic ductal leakage by the posterior wall of the stomach, the transverse colon, and the mesocolon, leading to the formation of a pseudocyst [4]. The rupture of the pseudocyst into the peritoneal cavity or the pleural cavity can result in ascites or pleural effusions, respectively [5]. Depending on the size of the pseudocyst, the resulting pancreatic ascites and pleural effusions can be moderate to severe [8]. In chronic pancreatitis, the leakage of pancreatic secretions may be only partially walled off, in which case, the resulting pseudocyst can cause persistent leakage into the peritoneal cavity or the pleural cavity [4]. In some instances, chronic pancreatic inflammation can disrupt the pancreatic duct, leading to the formation of an internal pancreatic fistula into the peritoneal cavity or the pleural cavity [9]. While the disruption of the pancreatic duct anteriorly leads to the formation of pancreatic ascites, the disruption of the pancreatic duct posteriorly results in pancreatic secretions tracking through the retroperitoneum into the mediastinum via the aortic and esophageal hiatus, leading to pancreatic-pleural fistula and pancreatic-pleural effusion [7].

Although a pancreatic-pleural fistula was a consideration in our patient, abdomen CT and MRCP showed no evidence of this condition. The complete resolution of the pleural effusion following chest drainage with no further recurrence of pleural effusion made pancreatic-pleural fistula a less-likely diagnosis. Rupture of the pancreatic pseudocyst was also a consideration. However, the pancreatic pseudocyst in the head of the pancreas was stable compared to previous CT images, and there was an interval increase of distal pancreatic pseudocysts, making pseudocyst rupture a less-likely cause of the black pleural effusion.

In our patient, the development of acute pancreatitis from chronic alcohol abuse with a background of pseudocysts and chronic pancreatitis became the major trigger for the formation of pancreatic ascites [10]. The ascitic fluid traversed from the peritoneal cavity to the pleural cavity through the fenestrations in the diaphragm [11]. Once the amylase-rich pancreatic fluid entered the pleural cavity, it caused further inflammation with reactive effusion, resulting in a massive left-sided pleural effusion [12]. The grossly elevated pleural fluid amylase confirmed the pancreatic origin of the pleural effusion.

Endoscopic retrograde cholangiopancreatography (ERCP) and MRCP are used to identify peripancreatic collections, pancreatic stones, pancreatic pseudocysts, and pancreatic-pleural fistula and to localize the site of leakage in the pancreatic duct. ERCP has the advantage of both diagnostic and therapeutic intervention [13]. However, one of the limitations of ERCP is that since it is an invasive procedure, the visualization of the pancreatic duct anatomy and the site of the pancreatic duct leak is difficult in the presence of strictures [5]. Conversely, the advantage of MRCP is that since the pancreatic duct and the fistula are displayed as high-signal-intensity structures, MRCP helps demonstrate the internal pancreatic fistula and pancreatic-pleural fistula accurately [5]. Through the performance of helical CT and MRCP in combination, the exact site of the rupture of the pancreatic duct could be observed in 94% of cases [5]. Thus, a combination of imaging modalities helps improve the accuracy of the findings [14]. In our patient, the results of the combination of abdomen CT and MRCP obviated the need for ERCP in planning the treatment strategy for the management of acute pancreatitis and its complications, namely, pancreatic ascites and black pleural effusion.

Patients with pancreatic ascites and pleural effusion from acute and chronic pancreatitis can be managed with conservative therapy and endoscopic and surgical therapy [15]. The critical steps in the conservative management of pancreatic ascites and pleural effusion are keeping the patient nil by mouth, providing nutrition through nasojejunal feeds, using somatostatin or octreotide to decrease pancreatic secretion, and draining pleural fluid using tube thoracocentesis [5,15]. In patients who do not respond to conservative therapy, the next step should be taken-either endoscopic or surgical therapy [15].

ERCP is a crucial step in the planning of endoscopic therapy [15]. Endoscopic therapy involves pancreatic sphincterotomy and transpapillary pancreatic stenting across the site of the pancreatic ductal leakage [16]. The pancreatic stenting helps reduce the high-pressure gradient at the pancreatic sphincter, which results in the flow of pancreatic secretions along the least resistance path into the duodenum, thereby facilitating the healing of the site of the leakage [16]. Patients who fail to improve with endoscopic therapy would require surgery [15].

The main indication for surgical treatment is the failure to improve with conservative and endoscopic therapies. The surgical intervention is planned considering the anatomy and site of the pseudocyst [17]. This intervention includes internal drainage, excision, and external drainage [18]. Commonly performed internal drainage surgeries include cystogastrostomy, cystoduodenostomy, and cystojejunostomy [18]. Cystogastrostomy is indicated for pseudocysts adherent to the posterior gastric wall. Cystoduodenostomy is indicated for pseudocysts located in the head and uncinate of the pancreas [18]. Roux-en-Y cystojejunostomy is usually performed for all types of cysts. Indications for surgical excision of the pseudocyst are painful chronic pancreatitis, multiple pseudocysts, gastrointestinal bleeding from pseudoaneurysm, the obstruction of common bile duct or duodenum, cystic neoplasia, splenic vein involvement, and technical inability to drain a pseudocyst located in the uncinate [18]. External drainage is indicated for immature cysts with infected contents and for ruptured cysts [19]. However, external drainage is associated with a high recurrence rate [4].

An episode of acute pancreatitis complicated by pancreatic ascites resulted in a massive left-sided black pleural effusion in our patient [3]. However, there was a remarkable resolution in the massive pleural effusion with the use of tube thoracocentesis. The resolution of acute pancreatitis resulted in a remarkable improvement of pancreatic ascites through conservative therapy, obviating the need for ERCP and surgical intervention. Due to the absence of a pancreatic-pleural fistula, no further recurrence of pleural effusions was observed.

## Conclusions

A black pleural effusion resulting from acute pancreatitis with a background of pancreatic ascites and pancreatic-pleural effusion is an extremely rare finding. Acute pancreatitis should be part of the differential diagnosis of the black pleural effusion, especially when the pleural fluid analysis shows elevated amylase and lipase levels. Black pleural effusion is a rare and unfamiliar type of pleural effusion that may indicate an underlying critical disease. Hence, a comprehensive evaluation is essential to diagnose the underlying etiology and initiate appropriate management in order to avoid fatal complications.
